# Anticipating manic and depressive transitions in patients with bipolar disorder using early warning signals

**DOI:** 10.1186/s40345-022-00258-4

**Published:** 2022-04-09

**Authors:** Fionneke M. Bos, Marieke J. Schreuder, Sandip V. George, Bennard Doornbos, Richard Bruggeman, Lian van der Krieke, Bartholomeus C. M. Haarman, Marieke Wichers, Evelien Snippe

**Affiliations:** 1 Department of Psychiatry, Rob Giel Research Center, University of Groningen, University Medical Center Groningen, PO Box 30.001, 9700 RB Groningen, The Netherlands; 2 Department of Psychiatry, Interdisciplinary Center Psychopathology and Emotion Regulation (ICPE), University of Groningen, University Medical Center Groningen, Groningen, The Netherlands; 3 Lentis Research, Lentis Psychiatric Institute, Groningen, The Netherlands; 4 Department of Psychiatry, University Medical Center Groningen, University of Groningen, Groningen, The Netherlands; 5 Present Address: Department of Computer Science , University College London , London, United Kingdom

**Keywords:** Early warning signals, Dynamical systems, Critical transitions, Bipolar disorder, Ecological momentary assessment, Experience sampling methodology, Complexity, Early detection, Smartphone, Mobile Health, Single-subject

## Abstract

**Background:**

In bipolar disorder treatment, accurate episode prediction is paramount but remains difficult. A novel idiographic approach to prediction is to monitor generic early warning signals (EWS), which may manifest in symptom dynamics. EWS could thus form personalized alerts in clinical care. The present study investigated whether EWS can anticipate manic and depressive transitions in individual patients with bipolar disorder.

**Methods:**

Twenty bipolar type I/II patients (with ≥ 2 episodes in the previous year) participated in ecological momentary assessment (EMA), completing five questionnaires a day for four months (*Mean* = 491 observations per person). Transitions were determined by weekly completed questionnaires on depressive (Quick Inventory for Depressive Symptomatology Self-Report) and manic (Altman Self-Rating Mania Scale) symptoms. EWS (rises in autocorrelation at lag-1 and standard deviation) were calculated in moving windows over 17 affective and symptomatic EMA states. Positive and negative predictive values were calculated to determine clinical utility.

**Results:**

Eleven patients reported 1–2 transitions. The presence of EWS increased the probability of impending depressive and manic transitions from 32-36% to 46–48% (autocorrelation) and 29–41% (standard deviation). However, the absence of EWS could not be taken as a sign that no transition would occur in the near future. The momentary states that indicated nearby transitions most accurately (predictive values: 65–100%) were full of ideas, worry, and agitation. Large individual differences in the utility of EWS were found.

**Conclusions:**

EWS show theoretical promise in anticipating manic and depressive transitions in bipolar disorder, but the level of false positives and negatives, as well as the heterogeneity within and between individuals and preprocessing methods currently limit clinical utility.

**Supplementary Information:**

The online version contains supplementary material available at 10.1186/s40345-022-00258-4.

## Introduction

A major challenge in psychiatry is to timely identify impending psychopathological episodes for individual patients. Until now, research has mostly focused on group-level retrospective risk factors (Meter et al. 2016), which unfortunately say little about *which* individual patient will relapse *when.* However, rapid technological advances have enabled patients to easily monitor their mood and symptoms in real-time, opening the door to prospective and personalized anticipation of clinically relevant symptom changes in the near future (Dunster and Swendsen 2020). Such early identification of episodes might be especially relevant for patients with bipolar disorder (BD), who experience frequent and disruptive depressive and manic episodes, and whose treatment is strongly focused on episode recognition (Michalak et al. 2006). Now that intensive longitudinal monitoring through smartphones has become increasingly feasible (Vachon et al. 2019), the field is in need of tools to utilize these data to anticipate future increases in psychopathological symptoms.

Principles derived from complex dynamical systems theory may provide such techniques. In complex dynamical systems, abrupt transitions to alternative states are anticipated by increasing instability of the system. This instability may be reflected by critical fluctuations and critical slowing down (Scheffer et al. 2009). Critical slowing down means that, as a system approaches a transition, it gets increasingly slow in recovering from minor perturbations (Scheffer et al. 2012). Critical slowing down manifests in patterns in the dynamics of time series data, including rising autocorrelation (i.e., the system’s current state increasingly predicts its next state) and rising variance (i.e., the system’s current state shows increasing fluctuations) (Scheffer et al. 2009, 2012). Because critical slowing down may occur prior to transitions in a system, rising autocorrelation and variance have been termed early warning signals (EWS). EWS have been shown to precede transitions in a wide variety of systems, such as climate change (Dakos et al. 2008), starlight shifts (George et al. 2020), and animal extinction (Drake and Griffen 2010).

Researchers increasingly recognize that psychopathology might also behave as a complex dynamical system (Cramer et al. 2016; Hayes and Andrews 2020). This provides a novel angle for understanding and anticipating large shifts between alternative stable states, such as bipolar and manic states. These shifts, called transitions, may result from impactful shocks (*e.g.,* loss of a loved one), rising noise (*e.g.,* being exposed to more frequent daily events due to exposure therapy), or from accumulating instability (which, in mathematic terms, can be considered a fold bifurcation). The latter possibility attracted much attention, as it means that transitions (*e.g.,* between depressed and manic states) may be anticipated by EWS. These EWS reflect rising instability in complex dynamical systems, and can be inferred from the dynamics of momentary affective and symptomatic states (Nelson et al. 2017; Olthof et al. 2020a; Bury 2020). These states can be assessed through ecological momentary assessment (EMA), with which patients frequently monitor their affect and symptoms in daily life on their smartphones. In psychopathology, rising autocorrelations in momentary states (e.g., affect, stress, worry) indicate higher carryover of one’s affective state from one moment to the next. This means that the effects of perturbations (e.g., stressful events) linger longer (Kuppens et al. 2010). Rising trends in variance mean that perturbations have an increasingly strong impact on one’s momentary state. Unlike rising autocorrelations, which exclusively relate to critical slowing down, rising variances have been related to both critical slowing down and critical fluctuations (Olthof et al. 2020a). Preliminary research suggests that EWS in momentary states can indeed anticipate prospective transitions from a healthy to a depressed state in patients with major depressive disorder (Wichers et al. 2016, 2020; Cabrieto et al. 2018). This raises the question whether EWS could also signal not only impending depressive, but also manic transitions in BD patients.

For BD patients, a smart personalized prediction tool based on smartphone monitoring could revolutionize treatment. Patients and clinicians describe this as one of the greatest promises of e-mental health (Murnane et al. 2016; Saunders et al. 2017). EMA might fulfill a dual purpose in this regard: the monitoring itself might already increase awareness of mood transitions, whereas the gathered data can be analyzed to provide a personalized EWS-based alert system (Bos et al. 2020). Indeed, previous research suggests that mood regulation in BD may be conceptualized as governed by nonlinear complex dynamical systems, as evidenced by, for example, a study showing that mood dynamics in BD are driven by relaxation oscillations (Bonsall et al. 2015), and another study showing lower entropy of mood in BD patients than in healthy controls (Ortiz et al. 2015). Furthermore, at the group-level, critical slowing down in affect and physical activity has been found to predict mood worsening (Curtiss et al. 2019), and in simulated rest/activity (i.e., actigraphy) data, EWS could be detected prior to mood transitions (Bayani et al. 2017). A logical next step would be to examine in empirical data whether EWS in momentary states indeed anticipate manic and depressive transitions in BD patients.

Therefore, the present empirical and exploratory study is the first to investigate whether EWS precede mood transitions in BD patients and whether they might have clinical utility. This investigation relies on the assumption that transitions between depressed and manic states resemble critical transitions due to a fold bifurcation in a bistable system. To that end, we employed an exploratory replicated single-subject design in twenty BD patients who participated in EMA for four months. First, we investigated for each patient which EWS occurred in which momentary state. Second, we examined at the group level whether EWS improved the detection of impending manic as well as depressive transitions, and whether the absence of EWS could be taken to indicate a lack of impending transitions. Finally, we studied which momentary states constituted the best EWS for manic and depressive transitions. Taken together, these findings might provide an explorative, comprehensive investigation into whether EWS may indeed be used to signal upcoming transitions in BD.

## Methods

### Participants

For the present prospective observational cohort study, twenty patients with BD type I or II were included. To be included, patients had to: 1) be ≥ 18 years, 2) be diagnosed with and currently in treatment for BD type I/II, and 3) demonstrate high occurrence of manic and/or depressive episodes (≥ 2 in the previous year). Clinicians of two Dutch tertiary care institutions invited patients for the study until the intended cap of twenty participants was reached. Interested patients were invited to the research facility, where the study was explained in detail and informed consent was signed.

Twenty-eight patients were referred, two of whom were unreachable. Six patients declined participation during the first telephone call, expecting that study participation and the focus on mood would be too burdensome. This left twenty patients that started and finished the study. Although a seemingly small sample, it is relatively large for idiographic studies. Here, power depends not on the number of individuals, but rather on the number of assessments per individual (Zuidersma et al.^2020^). As such, a sample of twenty patients allows us to examine the relative robustness of EWS in improving the detection of transitions. The study was approved by the University Medical Center Groningen medical ethics committee (no 201501161).

### Study design

For four months, patients completed five EMA assessments daily: every three hours (time-contingent schedule), patients received a text message with a link to the EMA on their smartphone, which took approximately 1–2 min to complete. Patients chose their own start and end time and had one hour to complete each assessment. No reminder prompts were given. Furthermore, during the 4-month study period, patients completed weekly symptom questionnaires, which were to be completed within 24 h. Assessments were securely administered and stored via RoQua (www.roqua.nl) in patients’ electronic health records. Researchers contacted patients after the first three days of monitoring. Patients were also contacted if compliance was low, if they preferred regular contact, or if the weekly questionnaires indicated above-threshold symptoms. Participants were not offered financial compensation.

### Measurements

#### Manic and depressive transitions

Patients completed questionnaires on manic^1^ and depressive symptomatology weekly. For manic symptoms, this was the Dutch version of the Altman Self-Rating Mania Scale (ASRM; Altman et al. 1997), a 5-item scale with which patients rate their manic symptoms on 0–4 scale (sum score ranges 0–20). For depressive symptoms, the Dutch version of the 16-item Quick Inventory for Depressive Symptomatology Self-Report (QIDS-SR; Rush et al. 2003) was used. Patients score their depressive symptoms on a scale of 0–3 (sum score ranges 0–48). On both scales, a score of ≥ 6 has been found to be indicative of a potential manic or depressive episode in bipolar samples (Miller et al. 2009; Bernstein et al. 2010). A transition was therefore defined as a clinically relevant abrupt (i.e., within one week) ≥ 6-point increase in manic (ASRM) or depressive (QIDS-SR) symptoms, without such increases in the two weeks prior to the transition (see Additional file 1).

#### EMA items

The questionnaire (see Additional file 1) consisted of 29 items pertaining to momentary mood, symptoms, sleep, and activities. These items were based on previous EMA research (Knapen 2019; Krieke et al. 2016; Tyler et al. 2015) and interviews with three patients and a psychiatrist on relevant constructs for people with BD. For the calculation of EWS, we selected the 17 EMA items that assessed affect or symptoms on a continuous scale (i.e., ranging from 0 (“not at all”) to 100 (“very much”)).

#### Compliance

Two patients were part of a pilot and therefore completed three months of EMA monitoring. The eighteen other patients completed on average 18 weeks of monitoring (range = 16–32). Average compliance, calculated as the number of completed assessments divided by the number of assessments participants received, was 76% (491 assessments, *SD* = 137.8, range = 197–869).

### Data analysis

For each EMA momentary state, two EWS indicators were examined: rises in the autocorrelation at lag-1 and rises in the standard deviation as indicator of the variance. These EWS were estimated using a moving windows approach (Wicher et al. 2016; Dakos et al. 2012). Briefly, this involves iterative computation of EWS within segments (or windows) of the time series, for each individual and transition separately. With each iteration, the window slides one time point ahead until the transition point. Within every window, we computed the autocorrelation and standard deviation of each momentary state. This yielded a new time series for each EWS indicator, allowing us to examine whether rises in the indicator actually preceded an abrupt manic or depressive transition. Pre-processing and analysis of the data were performed in the statistical programming language R (Team RDC 2014). Results were visualized using the R-packages *ggplot2* (Wickham 2016) and *gridExtra* (Auguie 2017). Our code is available in the Additional file 2.

#### Pre-processing steps

Outliers were winsorized to minimize their influence on the results: this sets any values below the 5th percentile to the 5th percentile, and values above the 95th percentile to the 95th percentile. To ensure there were no trends in the mean over time (i.e., stationarity) (Dakos et al. 2012), and reduce the risk of false positives (Jäger and Füllsack 2019), the data were detrended by applying a Gaussian kernel smoothing function over the whole pre-transition period (Dakos et al. 2008; Lenton et al. 1962). This removes non-linear trends in EMA states over time, effectively preventing confounding between trends in autocorrelations/variances (EWS) and trends in mean levels. To inspect the impact of this detrending method on our findings, as sensitivity analyses, we also ran our analyses with a linear detrending method (i.e., removing a linear trend over time within each window; see Additional file 1). Agreement between the two detrending methods was calculated as Cohen’s kappa, which is a measure of interrater agreement (in this context, inter-method agreement) and can be interpreted as low (< 0.40), weak (0.40–0.59), moderate (0.60–0.79), or high (> 0.80) (McHugh 2012; Cohen 1960). Missing data were not imputed because this might result in spurious correlations (Dakos et al. 2012).

#### Window size

The window size is related to the timescale at which the system dynamics evolve and may affect results (Dakos et al. 2012). Therefore, sensitivity analyses were conducted to investigate the effects of windows of one, two or three weeks. We ensured that each window included an equal number of weekend days, thereby negating their effects on the results (Bayani et al. 2017). The sensitivity analyses indicated small differences for different window sizes (see Additional file 1), but results were largely robust. Therefore, based on suggestions on how bipolar dynamics develop in patients with high mood instability (Bonsall et al. 2012), we opted for a window size of two weeks (i.e., 70 observations). This means that, for a transition that happened at the 150th observation, 150–70 = 80 windows could be fitted, hence yielding 80 estimates of autocorrelations/standard deviations. The average transition happened later in time, and therefore, there were on average 168 windows (*SD* = 99, range = 35–415) per transition.

#### Calculation of EWS

First, within each window, the autoregressive coefficient was calculated at lag-1 over the residuals obtained after detrending (Dakos et al. 2012). The autocorrelation thus indicates how well a momentary state (e.g., feeling cheerful) predicts itself three hours later. Overnight lags were prevented by not computing the lagged association between the evening observation and the next morning observation when calculating the autocorrelation within each window. Second, variance was estimated from the standard deviation^2^ over the residuals within each window (Dakos et al. 2012). A rising standard deviation indicates that someone’s momentary state varies more widely around the mean over time.

#### Significance testing

The above approach resulted in a new time series data set for every individual, with separate estimates of the autocorrelation and standard deviation. To test whether each EWS indicator significantly increased, we calculated the Kendall correlation coefficient (τK) over this data set (Dakos et al. 2012), in the two weeks prior to the transition (using the R-package *Kendall* (Kendall 2011)). If there were fewer than two weeks of observations prior to the transition, we used the available data to compute EWS. A significant positive correlation indicates that the EWS indicator significantly rose prior to a transition. The value of Kendall’s tau was taken to reflect the strength of the EWS. Since the moving windows use overlapping data, we corrected for this dependency between nearby windows by applying the Hamed-Rao correction (Hamed and Rao 1998). Furthermore, we corrected for multiple testing by using the false discovery rate (FDR) approach as proposed by Benjamini and Hochberg (Benjamini and Hochberg 1995). This ensures that, across all tests performed within a single patient, the probability of false positives is 5%.

#### Predictive value

To gain insight into the predictive utility of EWS for detecting mood transitions, positive and negative predictive values (PPV and NPV respectively) were calculated (Altman and Bland 1994). The PPV and NPV are based on the sensitivity (proportion of true positives, where EWS preceded transitions) and specificity (proportions of true negatives, where the absence of EWS indicates the absences of transitions). Predictive values indicate to what extent (i) the presence of EWS increases the likelihood of detecting a transition (PPV) and (ii) their absence increases the likelihood of detecting that *no* transition will take place (NPV). PPV and NPV were calculated for both EWS indicators averaged across momentary states, as well as for each momentary state separately. Predictive values follow the below formulas, using the sensitivity and specificity of a particular momentary state (i) for detecting transitions (t) towards either mania or depression:

$$PPV_{i,t} = \frac{{sensitivity_{i,t} *prevalence_{t} }}{{sensitivity_{i,t} *prevalence_{t} + \left( {1 - specificity_{i} } \right)*\left( {1 - prevalence_{t} } \right) }}$$

$$NPV_{i,t} = \frac{{specificity_{i} *(1 - prevalence_{t} )}}{{(1 - sensitivity_{i,t} )*prevalence_{t} + specificity_{i} *\left( {1 - prevalence_{t} } \right) }}$$

Higher predictive values indicate higher accuracy of EWS in detecting transitions. A PPV of 100% indicates that an EWS has no false positives, whereas a NPV of 100% indicates an EWS has no false negatives. The PPV and NPV demonstrate whether EWS improve the accuracy of detecting transitions above the general prevalence of transitions in our sample, and should thus be higher than the average transition prevalence. This transition prevalence was given by the proportion of individuals who experienced a manic transition (i.e., 7 out of 22, or 32%), a depressive transition (i.e., 8 out of 22, or 36%), or no manic (68%) or depressive (64%) transition. See Additional file 1 for a step-by-step calculation of predictive values and sensitivity and specificity for each EWS.

To estimate false positives, EWS were also computed for individuals without a transition. For them, we selected a period of approximately 245 observations (i.e., mean number of observations in the pre-transition period of transitioning patients), without any changes of  ≥ 4 on the ASRM and QIDS-SR. This was possible for seven out of nine non-transitioning patients.

## Results

### Sample characteristics

Demographic and clinical characteristics are depicted in Table 1. Of the twenty patients, eleven reported an abrupt manic or depressive mood transition, four of whom reported two transitions. Notably, during depressive transitions, all patients reported subthreshold mania scores. During manic transitions, all but two patients reported above threshold scores for depression.

**Table 1:** Demographic and clinical characteristics

	Full sample (N = 20)	Patients with transition (N = 11)	Patients without transition (N = 9)
Gender (N)			
Male	4	3	1
Female	16	8	8
Age (N)			
20–35 years	9	4	5
36–50 years	8	5	3
51–65 years	3	2	1
Education level (N)			
Higher education	9	6	3
Secondary education	5	2	3
Secondary vocational education	3	1	2
Pre-vocational education	3	2	1
Years since bipolar disorder diagnosis (*M, SD)*	6.4 (6.3)	5.0 (5.8)	8.2 (6.8)
Years in treatment (*M, SD*)	10.6 (8.8)	10.1 (8.5)	11.27 (9.7)
Bipolar disorder diagnosis (N)			
Bipolar disorder type I	9	6	5
Bipolar disorder type II	11	5	4
Comorbid diagnoses (N)			
No comorbid Axis I/II disorder	12	5	7
Attention Deficit/Hyperactivity Disorder	1	1	0
Autism Spectrum Disorder	1	1	0
Sleep disorder	1	1	0
Alcohol/drug dependence	1	0	1
Personality disorder	6	5	1
Medication use at study start (N)			
None	2	1	1
Amphetamine	1	1	0
Anti-epileptic	10	8	2
Atypical antipsychotic	10	5	5
Benzodiazepine	9	6	3
Thyreomimetica	2	0	2
Lithium	5	0	5
Monoamine oxidase inhibitor	3	3	0
Selective serotonin reuptake inhibitor	4	2	2
Tricyclic antidepressant	1	1	0
Transitions^1^ (N)			
To a manic episode		7	
To a depressive episode		8	
Symptom increase in week of transition (*M, SD*)			
Transition to a manic episode (ASRM)		6.7 (1.5)	
Transition to a depressive episode (QIDS-SR)		11.3 (5.8)	
Manic and depressive symptom levels (*M, SD*)			
During manic periods (ASRM ≥ 6)	9.0 (3.3)		
During depressed periods (QIDS ≥ 6)	11.5 (4.7)		
During nonmanic periods (ASRM < 6)	1.3 (1.6)		
During nondepressive periods (QIDS < 6)	3.3 (1.3)		
Episode duration after transition in weeks (*M, SD*)			
Manic episode		1.9 (0.9)	
Depressive episode		2.6 (3.1)	
Compliance to EMA (*%*)	75.9	74.6	77.6

^1^Four patients reported two transitions, the remaining seven patients reported one transition

*ASRM * Altman Self-Rating Mania Scale, *M* mean, *N* number, *QIDS-SR* Quick Inventory of Depressive Symptomatology Self-Report, *SD* standard deviation

### A clinical illustration

**Fig. 1:**
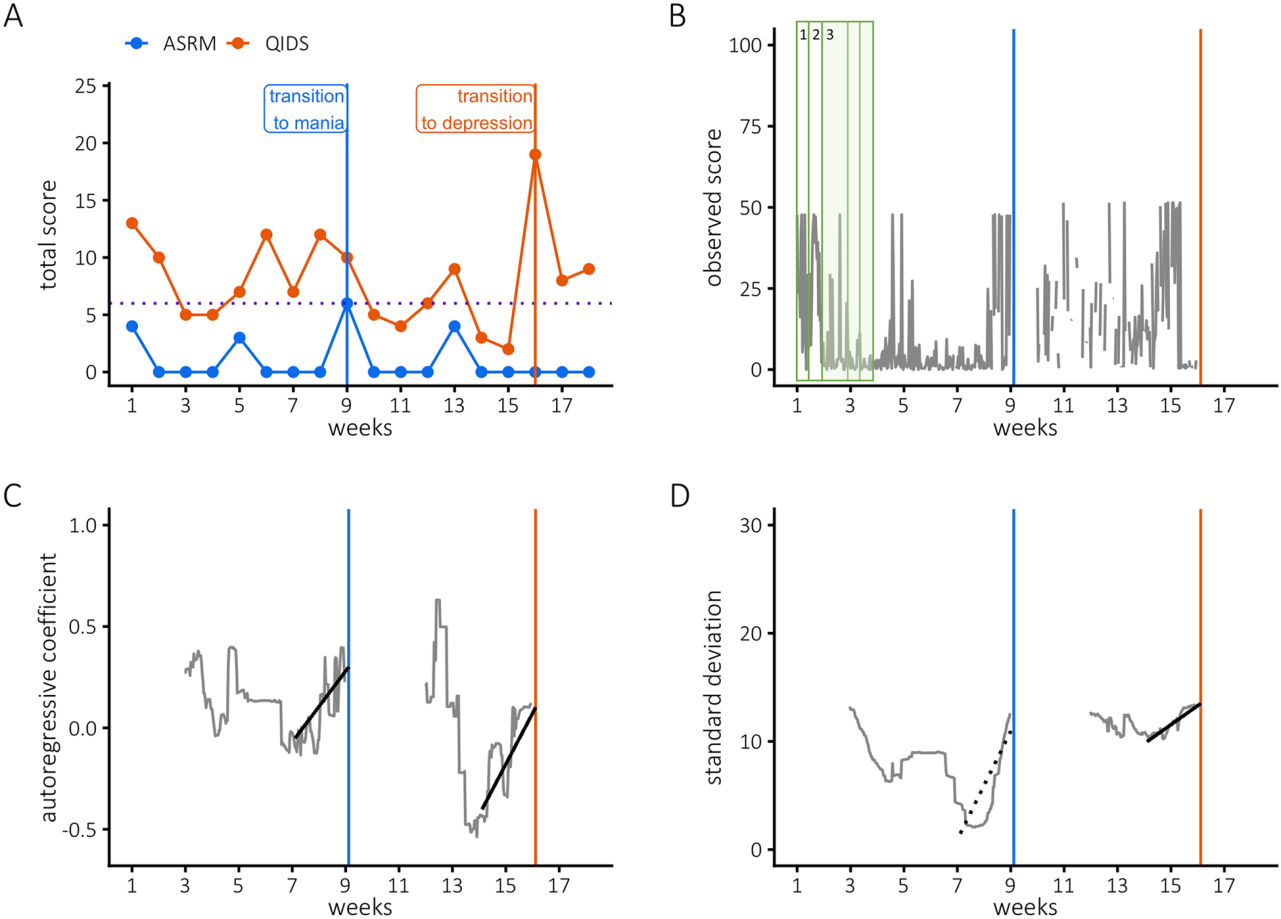
An illustration of early warning signals in one individual (ID6) in the item “I feel extremely well”. A depicts weekly manic (Altman Self-Rating Scale, ASRM, blue) and depressive (Quick Inventory of Depressive Symptomatology, QIDS-SR, red) symptom scores. At week 8 and 15, she reports an abrupt transition to a manic and depressive episode, respectively. Figure 1B visualizes her raw ecological momentary assessment (EMA) scores on “feeling extremely well”. Higher scores indicate she is feeling more euphoric. We iteratively fitted windows containing two weeks of observations (green rectangles). These windows slided through the time series, meaning that the first window contained observations 1–70, the second window contained observations 2–71, etc. Note that the windows in the figure solely serve to illustrate the main idea behind the analyses. Within each window, we computed the autocorrelation and standard deviation as early warning signals (EWS). This yielded surrogate time series of the autocorrelation and standard deviation. As shown in Fig. 1C, significant EWS were found prior to the manic transition (Kendall’s Tau = .54, *corrected p* < .001) as well as the depressive transition (Kendall’s Tau = .68, *corrected p* < 0.001). Figure 1D shows an EWS in the standard deviation prior to the depressive transition (Kendall’s Tau = .75, *corrected p* < 0.001), but not prior to the manic transition (Kendall’s Tau = .50, *corrected p* = 0.07)

We will first present a clinical case example to illustrate EWS in an individual patient (ID6): a 27-year old woman diagnosed with BD type II. She reported both a manic and a depressive transition on the weekly ASRM and QIDS-SR (Fig. 1A). Her weekly symptom scores had been stable in the previous weeks. Note that this would imply similar stability in her EMA momentary states until the transitions.

Figure 1B shows her EMA observations for the state *‘extremely well’*. Higher scores indicate she is feeling more euphoric. It is difficult to distill clear patterns from these data; we see a lot of moment-to-moment variation, and more missed assessments after the manic transition. Figure 1C shows significant EWS in the autocorrelation for “*extremely well*” prior to both transitions. This means that, within the two weeks before reporting a manic and depressive shift, her euphoria increasingly lingered over time. Figure 1D shows an EWS in the standard deviation for the depressive transition, but not for the manic transition, indicating that her euphoria varied more widely prior to the depressive transition.

### Early warning signals prior to transitions

All transitions, both depressive and manic, were preceded by at least one EWS (i.e., a significant rise in the autocorrelation or standard deviation) in at least one of the EMA momentary states. Regarding the autocorrelation, on average, most EWS were found prior to manic transitions (*M* = 5.6) versus depressive transitions (*M* = 4.5) and non-transitions (*M* = 4.3). The standard deviation also yielded the most EWS prior to manic transitions (*M* = 6.7), versus depressive transitions (*M* = 2.8) and non-transitions (*M* = 5.3).

### Individual differences

**Fig. 2:**
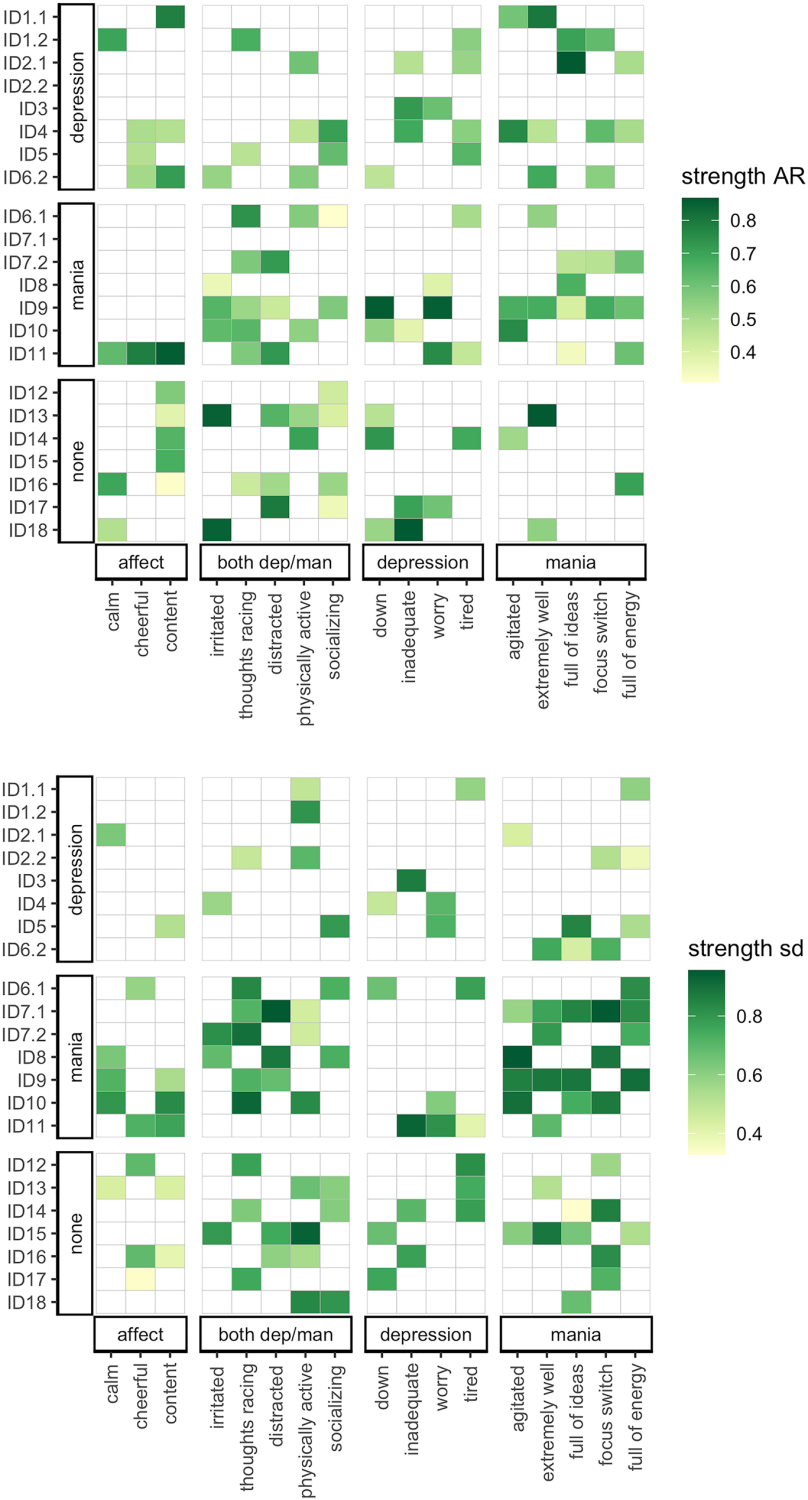
Individual differences in the type and strength of the early warning signal. The x-axis represents each EMA momentary state, the y-axis each transition. Note that four individuals had two transitions (denoted by digits, with the lowest digit corresponding to the first transition). EWS were detected using moving window analyses (window = 2 weeks). To facilitate interpretation, the EMA momentary states were assigned to summary categories based on hypothesized underlying constructs. A colored block indicates that the EWS was significant for that transition. The color intensity indicates the strength of the EWS: the more intense the color, the stronger the EWS. Strength of the EWS was operationalized as the value of Kendall’s tau. *Abbreviations:* *AR* autocorrelation at lag-1, *EMA* ecological momentary assessment, *EWS* early warning signal, sd = standard deviation

Large individual differences were found in the presence, type, and strength of EWS momentary states (see Fig. 2). For example, ID4 showed EWS in the autocorrelation for ten momentary states prior to a depressive transition, whereas ID2 reported none prior to their second depressive transition. Whereas the autocorrelation of ‘*down’* was a particularly strong depressive EWS for ID9, this EWS was not found in any of the seven other transitions. For the four patients that reported two transitions, EWS often did not replicate. Exceptions include the autocorrelations of *‘extremely well’* (ID6) and *‘physically active’* (ID6), and the standard deviations of ‘*physically active’* (ID1 and ID7) and *‘extremely well’*, *‘full of energy’*, *‘physically active’*, and *‘racing thoughts’* (ID7).

### Anticipatory value of early warning signals

**Fig. 3:**
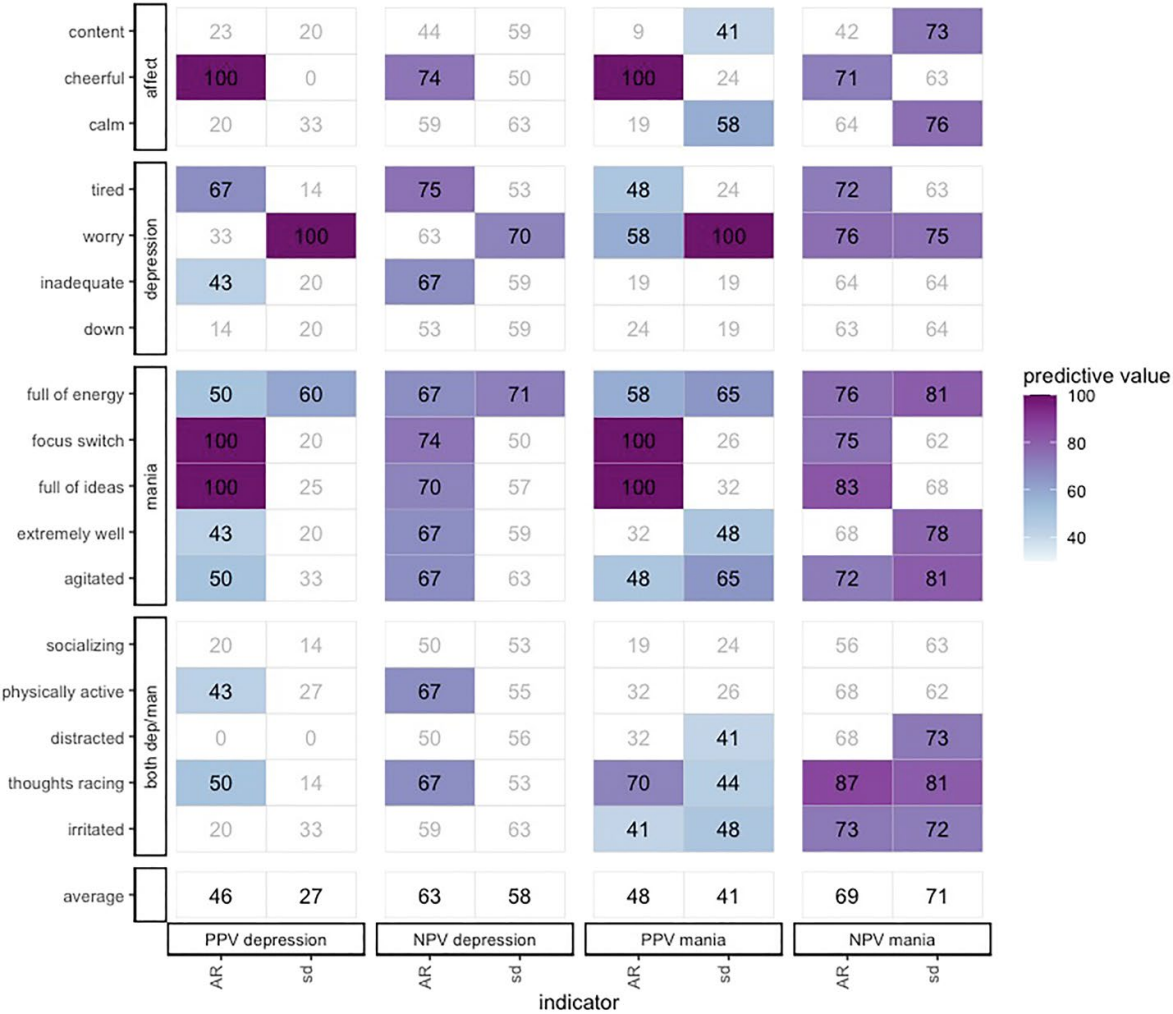
Positive and negative predictive values for each early warning signal. The y-axis represents each momentary state, the x-axis the positive (PPV) and negative (NPV) predictive value, separated for manic and depressive transitions and for the two early warning signals (EWS) indicators: the autocorrelation (AR) and standard deviation (SD). The predictive values can be compared against the prevalence of the transition: the proportion of manic (32%), depressive (36%), or no transitions (68% for mania and 64% for depression). White tiles indicate that this EWS did not improve the detection of a transition above the prevalence of that transition. The color indicates the magnitude of the predictive value for that EWS: the more intense the color, the higher the predictive value. To facilitate interpretation, the EMA momentary states were assigned to summary categories based on hypothesized underlying constructs

PPVs and NPVs indicate, at the group-level, to what extent (i) the presence of EWS increases the likelihood of detecting a transition (PPV) and (ii) their absence increases the likelihood of detecting that *no* transition will take place (NPV). In general, average PPVs and NPVs indicate that EWS were better in signaling the presence of transitions than their absence (see Fig. 3). The agreement between the autocorrelation and standard deviation was relatively low for transitions towards depression (overlap of 7%) and somewhat higher for transitions towards mania (overlap of 28%; see Additional file 1). Supplementary analyses, where we used linear within-windows detrending instead of non-linear detrending over the whole time series, yielded similar findings on average PPVs and NPVs (see Additional file 1).

Regarding depressive transitions, the average PPV indicates that if at least one EWS in the autocorrelation was detected, the average probability of anticipating a depressive transition increased from 36% (prevalence) to 46%. However, if EWS were absent, the probability of correctly inferring *no* depressive transition did not improve (NPV = 63% versus 64% prevalence). The standard deviation was not an accurate EWS for depressive transitions, with a PPV of 27% and a NPV of 58%.

For manic transitions, the autocorrelation was again slightly more accurate in signaling transitions. Here, EWS improved the probability of correctly inferring a manic transition from 32% (prevalence) to 48% (autocorrelation) or 41% (standard deviation). However, the NPV demonstrates that the probability of inferring *no* manic transition in the absence of EWS improved only slightly from 68% (prevalence) to 69% (autocorrelation) or 71% (standard deviation).

### Anticipatory value of specific EMA momentary states

Figure 3 demonstrates the PPVs and NPVs for all momentary states separately. Four momentary states had a PPV of 100% for both depressive and manic transitions: ‘*cheerful’* (autocorrelation), *‘ability to focus/switch’* (autocorrelation), ‘*full of ideas’* (autocorrelation), and *‘worry’* (standard deviation), indicating they were never found for patients without a transition. The NPVs of these momentary states ranged from 70 to 83%. Kappa indicated moderate to strong agreement between detrending methods for *'full of ideas'* and '*worry'*, but not for ‘*cheerful’* and ‘*ability to focus/switch’*, indicating that only the former two emerged as relatively robust EWS across detrending methods (see Additional file 1). These momentary states might thus signal an impending transition without specifying its nature (depressive or manic).

Other momentary states were found to specifically improve the detection of either depressive or manic transitions, as indicated by an average PPV and NPV above 70%. For depressive transitions, the only momentary state yielding such EWS was *‘tired’* (autocorrelation). However, Kappa indicated low agreement between both detrending methods for EWS in this momentary state. For manic transitions, EWS with the highest PPV and NPV were found in ‘*racing thoughts*’ (autocorrelation), ‘*agitated’* (standard deviation), and ‘*full of energy’* (standard deviation). However, only for ‘*agitated’* Kappa indicated strong agreement.

Some momentary states always had a PPV and NPV below the prevalence, indicating that, although these EWS were detected for some individuals, at the group level, they did not improve the detection of manic or depressive symptoms. This was the case for *‘socializing’* and ‘*down’*. Similarly, *‘content’* (autocorrelation) demonstrated low PPV and NPV for manic transitions. For depressive transitions, three momentary states had a PPV of 0% because EWS were never detected prior to transitions: *‘distracted’* (autocorrelation and standard deviation) and *‘cheerful’* (standard deviation). Again, here, the agreement between detrending methods was low to moderate, meaning that the item-level results depend on the type of preprocessing that is used. Therefore, these results should be cautiously interpreted.

## Discussion

The present exploratory study investigated whether EWS in momentary affective and symptomatic states anticipate abrupt mood transitions in BD patients, and whether EWS might have clinical utility. Results provide preliminary support that EWS can indeed be detected in momentary states that were collected by longitudinal smartphone monitoring. The presence of EWS increased the probability of detecting impending transitions, but their absence could not be taken as a sign that no transition would occur in the near future. That is, although several momentary states maximized the PPV, indicating that no false positives were found, no momentary state maximized the NPV, indicating the presence of false negatives (i.e., EWS were not always detected prior to transitions). Furthermore, momentary states differed in their predictive utility, which also depended on detrending method, and we found large inter- as well as intra-individual differences in the predictive capacity of EWS.

Several momentary states were more promising indicators of nearby depressive and manic transitions than others, and emerged robustly across detrending methods. Momentary states that signaled both manic and depressive transitions were *full of ideas* and *worrying*. Manic transitions were most often anticipated by EWS in *agitation.* Depressive transitions were not anticipated by EWS that were robust across detrending methods. This provides preliminary support to the hypothesis that transitions are best anticipated by EWS in momentary states matching the underlying psychopathology (Wichers et al. 2019). Importantly, not all momentary states constituted as accurate EWS, highlighting the importance of exploring individual momentary states.

Contrasting to group-level risk factors that offer little guidance on the timing of relapses for individual patients (Fisher et al. 2018), researchers expect EWS to be promising indicators of *when* individual patients experience symptom transitions (Wichers et al. 2016). Timely identification of manic and depressive transitions is paramount to BD treatment, but is complicated because patients usually only recognize them when episodes have already started (Murnane et al. 2016). Early detection using EWS inferred from smartphone data would enable clinicians to intervene early on, which could mitigate the severity and impact of ensuing episodes (Morriss 2002). Given this promise, thorough empirical investigation is warranted to test the clinical utility of EWS. However, our results show that such clinical utility, and thereby the implementation of EWS as clinical tools, is yet far away. Indeed, EWS in specific momentary states did not consistently precede transitions across individuals (false negatives), EWS were sometimes also found in patients without transitions (false positives), and EWS were dependent on detrending method. These false negatives and false positives, as well as the large heterogeneity in the types of EWS (across indicators, between and within individuals, and across preprocessing methods), complicate the clinical usage of EWS.

A likely explanation for false positives is that autocorrelations and variances may change for reasons other than critical slowing down—which confounds the evaluation of EWS. For instance, false positives may occur when the data show more noise or variability (Boettiger et al. 2013). Patients in our sample indeed showed a high frequency of transitions, similar to (ultra) rapid-cycling patients that experience stark fluctuations of mood most of the time (Kramlinger and Post 1996). It is also possible that patients experienced more events over time (*e.g.*, linked to remitting depression), causing a rising trend in variance that is not due to critical slowing down. Speculatively, a final explanation for the occurrence of false positives is that the individuals for whom we found these signals may have experienced a period of destabilization without tipping to the alternate state (Olthof et al. 2020b; Gelo and Salvatore 2016). Further, the absent agreement between the autocorrelation and standard deviation as indices of EWS suggests that dynamics other than critical slowing down, such as critical fluctuations, warrant further exploration.

Importantly, failing to anticipate actual transitions (false negatives) might be more problematic for clinical applications than the occasional false alarm. In the present study, EWS occurred more often in the standard deviation compared to the autocorrelation, but also more often resembled false alarms, meaning that the autocorrelation might be considered a more reliable indicator of impending transitions in bipolar disorder. This supports earlier studies in EWS, both in psychiatry (Curtiss et al. 2021) as well as in other fields (Dakos et al. 2012; Burthe et al. 2016). False negatives (absent alarms) may have various explanations. Firstly, the absence of EWS may be explained by the fact that most transitions are often governed by only a few variables (Maas and Molenaar 1992). Indeed, the momentary states that contained EWS differed between the individuals in our sample. Second, false negatives may have occurred because of the lack of stable mood episodes in the patients in our sample. That is, critical slowing down assumes transitions take place from one stable state to another, whereas the ‘stable’ state of patients in our sample may well be characterized by large mood instability (Helmich et al. 2021). Third, false negatives may be due the fact that the transitions we investigated might not necessarily match the type of transitions that are anticipated by critical slowing down (namely, transitions occurring through so-called zero eigenvalue bifurcations triggered by a minor perturbation). If transitions were for instance caused by a large, stressful event (*e.g.,* loss of a loved one), observing critical slowing down is less likely. Finally, false negatives could occur because the timescale of the EMA assessments (five/day) may not match the timescale at which critical slowing down takes place (Haslbeck and Ryan 2021).

What follows from this elaboration is that critical slowing down, and by virtue, EWS, are subject to a strict set of assumptions or requirements (*e.g.,* with respect to the variables that are monitored, the stability of mood states, the definition of transitions, and the resolution of EMA assessments). At present, it is still unknown whether the bipolar mood system meets these criteria. Hence, future research is necessary to delineate this system. Should we, for instance, attempt to anticipate distinct mood episodes, or rather mood instability in general (Hadaeghi et al. 2013)? The latter approach may be more promising given that many BD patients have mixed episodes, as also demonstrated by the patients in our sample. Thus, an interesting follow-up study would be to investigate EWS in more stable BD patients, who transition from stable euthymic states to manic or depressed states.

The present study is the first to investigate whether EWS anticipate transitions in BD patients in empirical data. Notably, previous studies have only examined autocorrelation and standard deviation as static indicators of future transitions (Bonsall et al. 2015; Curtiss et al. 2019; Ortiz and Alda 2018), whereas we have prospectively examined increases in these indicators to anticipate nearby transitions. Further, we used a personalized and idiographic approach that has been advocated to study within-person processes (Zuidersma et al. 2020). Other strengths include the relatively large sample (N = 20) for idiographic studies, in which each patient could be viewed as a replication of results in other patients, and the large number of observations per person. Furthermore, the diversity in momentary states under investigation might provide new suggestions for confirmatory research.

The present results should be viewed in light of several limitations. First, our sample was diverse, consisting of BD patients of both type I and type II, with different treatment regimens and comorbid (personality) diagnoses characterized by high mood variability or even rapid cycling, which might have obscured the relation between EWS and transitions. Our results may not generalize to BD patients who experience more distinct, stable episodes. Relatedly, it is possible that EWS are only useful for a specific subset of BD patients, which we could not address as a larger sample would be needed to do so. Second, EWS studies in other fields suggest that results may be dependent on the analytical decisions regarding window size, data detrending, and the period over which the rise is calculated (two weeks) (Dakos et al. 2012; Lenton et al. 1962). Third, the question remains whether the above-threshold scores on weekly self-report symptom questionnaires adequately reflect abrupt and clinically meaningful episodes. Fourth, our estimates of the prevalence of transitions should be interpreted tentatively given our sample size. However, the prevalence of transitions was quite high in this sample, rendering our approach to compare the predictive values against the prevalence rather conservative. Fifth, given that our study was exploratory in nature, results may not generalize to other samples or data sets in which different methods to study EWS are employed.

Finally, it is important to denote several limitations of EWS. These limitations are not specific to the current study per se, but rather, apply to the broader research into EWS. Firstly, autocorrelations and variances can only assess critical slowing down indirectly, which inevitably creates unreliability. Secondly, EWS are not as generic as typically supposed: these signals only occur if the right type of variable is assessed at the right frequency prior to the right type of transition (Boerlijst et al. 2013). What is “right” in the context of psychiatry is currently unknown—meaning that we do not know whether or under what circumstances psychiatry meets the criteria for detecting EWS. One goal for further research could therefore be to better define bipolar disorder as a complex dynamical system—e.g., in terms of the variables of which it consists, the type and number of stable states that it features, and in terms of the types of transitions between these states. A better understanding of the bipolar mood system will eventually help to determine if, when, and for whom EWS are clinically useful.

## Conclusions

To conclude, EWS can be detected prior to manic and depressive transitions in BD, but also yield false positives and negatives. As such, a complex dynamical systems approach is currently mostly relevant to enhance our understanding of the bipolar disorder mood system, but may have limited utility for anticipating manic and depressive transitions in treatment. Further research is necessary to further explore the utility of a complex dynamical system approach to BD, for instance by examining the (in)stability of depressive and manic episodes and further delineating the type of transitions between these episodes. This will ultimately help to determine if (and for whom) EWS indeed fulfill the clinical promise that they have been suggested to hold.

## Supplementary Information

**Additional file 1**. Supplementary Materials.

**Additional file 2**. R code for the analyses of this paper.

## Data Availability

The data that support the findings of this study are available from the corresponding author upon reasonable request.

## Abbreviations

ASRM: Altman Self-Rating Mania Scale
BD: Bipolar disorder
EMA: Ecological momentary assessment
EWS: Early warning signal(s)
NPV: Negative predictive value
PPV: Positive predictive value
QIDS-SR: Quick Inventory of Depressive Symptomatology

## Footnotes

1 Although N = 9 patients had BD type II, suggesting they experience hypomanic transitions, we refer to both hypomanic and manic transitions as ‘manic transitions’ for consistency.

2 The standard deviation is the square root of the variance. This transformation does not affect the rank correlation coefficient Kendall’s tau.
