# Supplementary material for: Anticipating manic and depressive transitions in patients with bipolar disorder using early warning signals
Source: Int J Bipolar Disord. 2022 Apr 9;10:12. doi: 10.1186/s40345-022-00258-4 (PMC8994809; doi:10.1186/s40345-022-00258-4)
Supplement: Supplementary file 1 — Additional file 1. Supplementary Materials. [file 40345_2022_258_MOESM1_ESM.docx]

Supplementary materials for:

Anticipating manic and depressive transitions in patients with bipolar disorder using early warning signals

RUNNING HEAD: EARLY WARNING SIGNALS IN BIPOLAR DISORDER

Fionneke M. Bos*^1,2^, Marieke J. Schreuder^1^, Sandip V. George^1^, Bennard Doornbos^3^, Richard Bruggeman^2^, Lian van der Krieke^1,2^, Bartholomeus C.M. Haarman^4^, Marieke Wichers^1^, Evelien Snippe^1^

^1^ University of Groningen, University Medical Center Groningen, Rob Giel Research Center, Department of Psychiatry, Groningen, The Netherlands

^2^ University of Groningen, University Medical Center Groningen, Interdisciplinary Center Psychopathology and Emotion Regulation (ICPE), Department of Psychiatry, Groningen, The Netherlands

^3^ Department of Specialized Training, Psychiatric Hospital Mental Health Services Drenthe, Outpatient Clinics, Assen, The Netherlands

Indicates both authors contributed equally.

*Corresponding author:

Fionneke Bos, M.Sc., Rob Giel Research Center, University of Groningen, University Medical Center Groningen, PO Box 30.001, 9700 RB, Groningen, The Netherlands. Phone: +31 50 361 5725, e-mail: [f.m.bos01@umcg.nl](mailto:f.m.bos01@umcg.nl).

**Contents**

1. [Overview of transition criteria](#_Overview_of_transition)
2. [All transitions per individual](#_All_transitions_per)
3. [Ecological momentary assessment (EMA) diary items](#_Experience_sampling_method)
4. [Results of the sensitivity analyses for the window size](#_Results_of_the)
5. [Calculation of positive and negative predictive values, sensitivity, and specificity](#_Calculation_of_positive)
6. [Effect of detrending](#_Effect_of_detrending)
7. [Agreement between EWS](#_Agreement_between_EWS)

## Overview of transition criteria

Supplementary Figure 1 demonstrates a flowchart of the criteria for transitions. Transitions had to meet three criteria. First, a symptom increase of >=6 on the Quick Inventory for Depressive Symptomatology Self-Report (QIDS-SR, depressive transitions) or the Altman Self-Rating Mania Scale (ASRM, manic transitions). Second, a stable two weeks prior to the increase, without increases of >=6. Third, and finally, at least three weeks of observations prior to the increase. In the figure, the left column denotes the number of patients meeting each of the criteria. The right column denotes the number of cases, and finally the number of manic and depressive transitions.

**Supplementary Figure 1. Overview of transition criteria.**

**
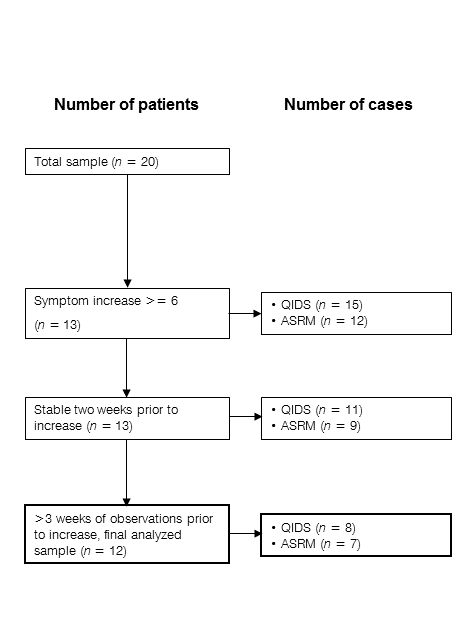
**

## All transitions per individual

In Supplementary Figure 2, all transitions per individual are depicted. Purple horizontal bars represent the stable period that was used to determine EWS.

**Supplementary Figure 2. All transitions per individual.**


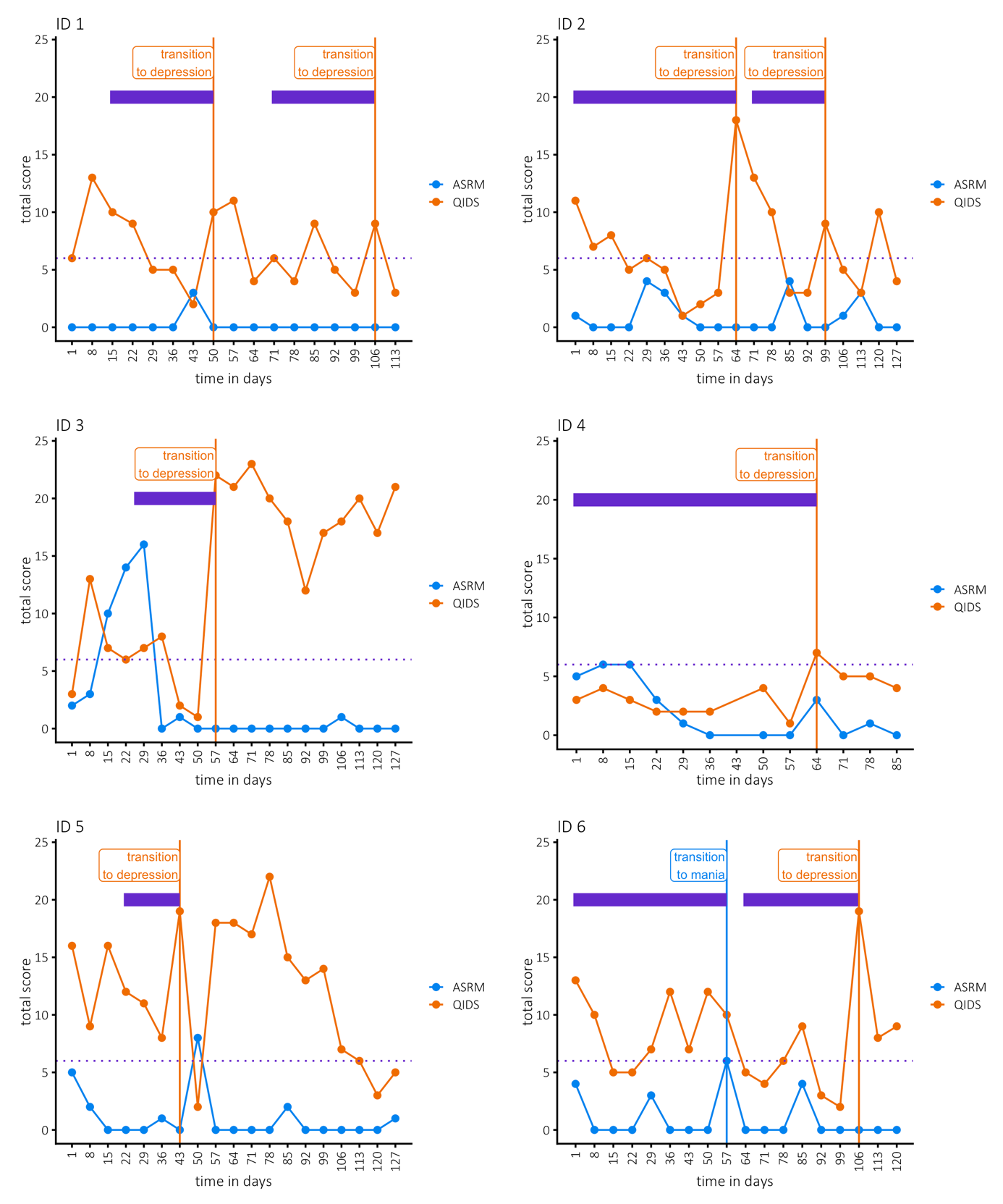


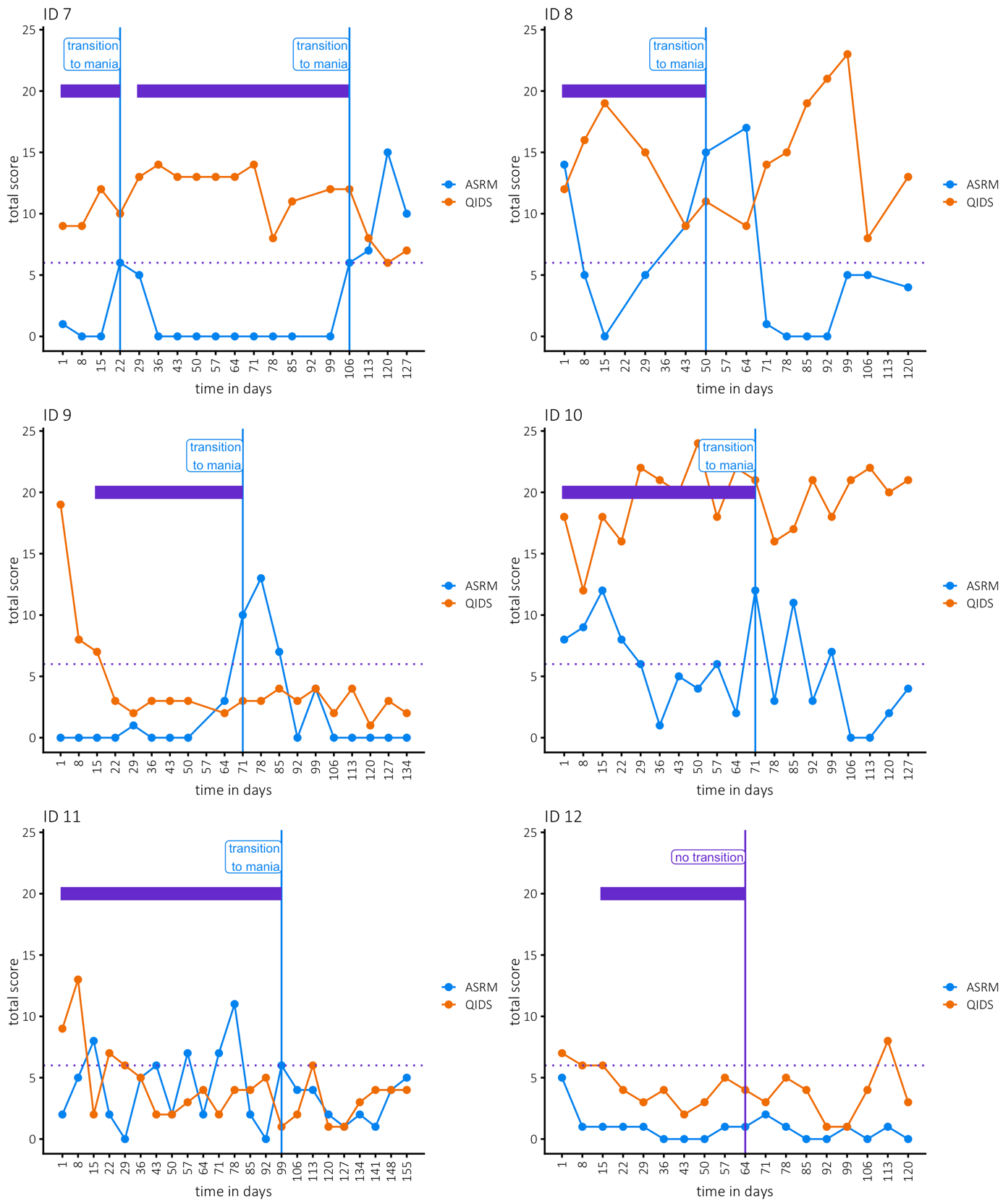


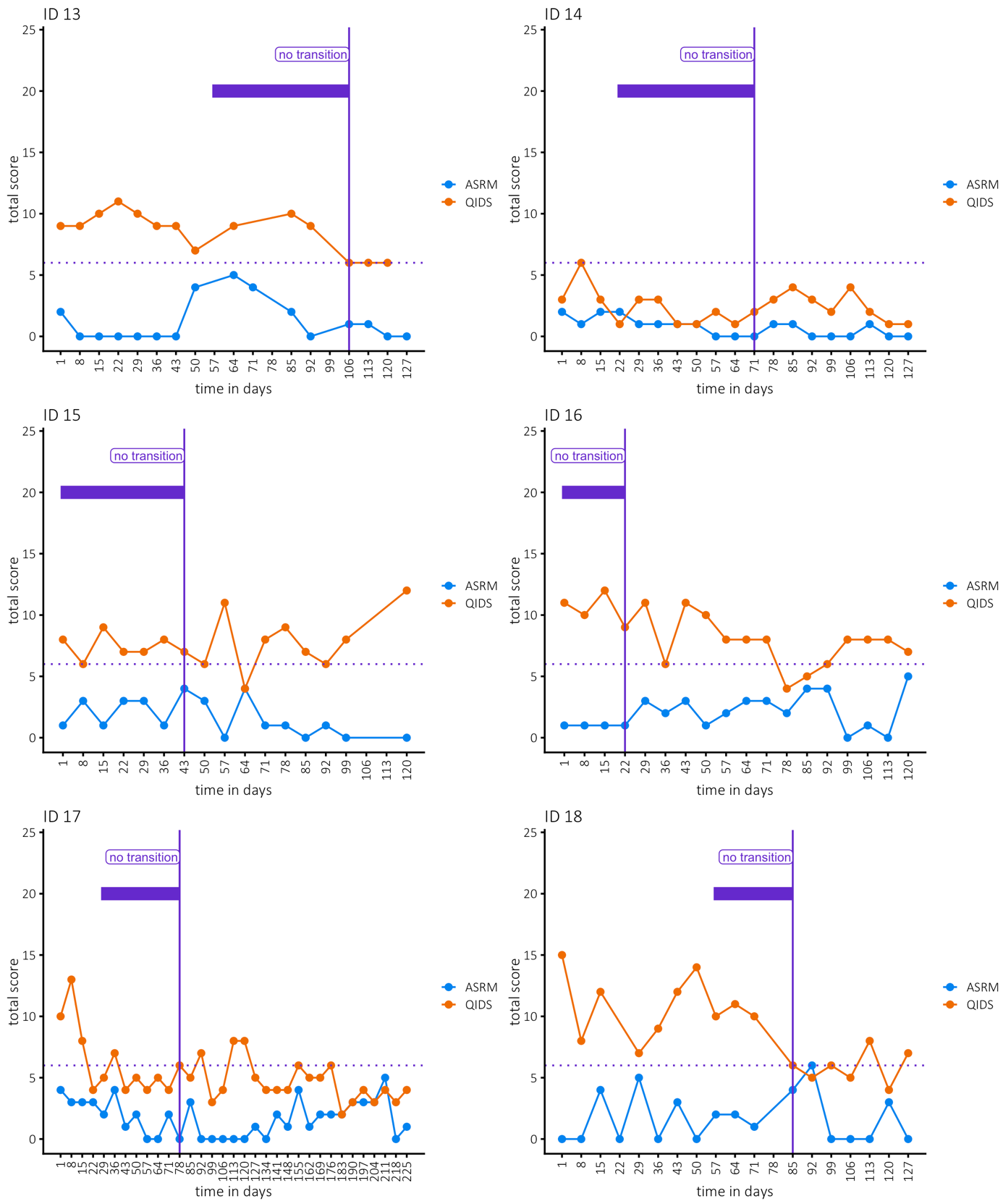


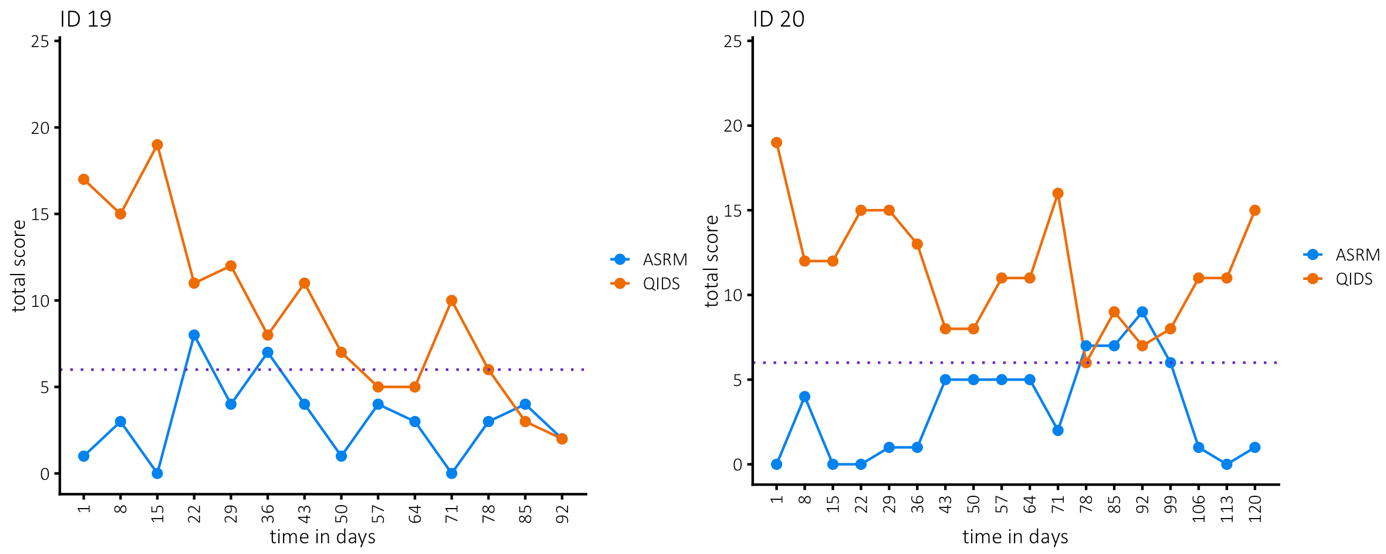


## Ecological momentary assessment (EMA) diary items

The item list (see Supplementary Table 1) was constructed in several steps. First, we identified relevant concepts for symptoms of bipolar disorder and searched the literature for EMA studies in patients with bipolar disorder (1-3). This yielded a first item list of 71 potentially relevant items. This list was then discussed in individual interviews with three patients and one psychiatrist. The items were finally selected on three criteria: 1) the patients and clinician recognized the item as signaling symptoms of either mania or depression; 2) the patients and the clinician felt comfortable with the formulation of the item (e.g., they could see themselves saying the sentence in daily life); 3) both the patients, the clinician, and the research team believed the item would vary meaningfully within participants. Items that were deemed relevant but too person-specific were put on a list for participants so they might select them for their personal question.

All items were obligatory, with the exception of the comment field at the end of the questionnaire (item 29). All items were assessed five times per day, with the exceptions of the items regarding sleep (item 2- 3), and the item regarding appointments (item 27). These items were only shown if participants answered ‘yes’ on item 1 or item 26. This way, we ensured that participants could still answer questions about their sleep, even though they might have skipped the first (few) assessments.

| **Supplementary Table 1. Ecological momentary assessment (EMA) diary items.** | | | | |  |
| --- | --- | --- | --- | --- | --- |
|  | **Dutch** | **English translation** | **Anchors (far left, middle, far right)** | **Which pompt(s)** | **Reason for exclusion** |
|  | Is dit de eerste meting die u invult vandaag? | Is this the first assessment you complete today? | - Yes - No | All five prompts |  |
|  | 1. Hoe lang heeft u geslapen? | How long did you sleep? | 0 – 12 hours | If answered ‘yes’ on item 1 | Assessed on different timescale, yielding too few data points for analysis |
|  | 1. De kwaliteit van mijn slaap was… | The quality of my sleep was… | Very bad – reasonably – very good | If answered ‘yes’ on item 1 | Assessed on different timescale, yielding too few data points for analysis |
|  | Ik voel me opgewekt | I feel cheerful | Not at all – reasonably – very much | All five prompts |  |
|  | Ik voel me neerslachtig | I feel down | Not at all – reasonably – very much | All five prompts |  |
|  | Ik voel me kalm | I feel calm | Not at all – reasonably – very much | All five prompts |  |
|  | Ik voel me gejaagd | I feel agitated | Not at all – reasonably – very much | All five prompts |  |
|  | Ik voel me bijzonder goed | I feel extremely well | Not at all – reasonably – very much | All five prompts |  |
|  | Ik voel me moe | I feel tired | Not at all – reasonably – very much | All five prompts |  |
|  | Ik voel me tevreden | I feel content | Not at all – reasonably – very much | All five prompts |  |
|  | Ik voel me geïrriteerd | I feel irritated | Not at all – reasonably – very much | All five prompts |  |
|  | Ik zit vol energie | I am full of energy | Not at all – reasonably – very much | All five prompts |  |
|  | Ik zie op tegen de rest van de dag | I dread the rest of the day | Not at all – reasonably – very much | All five prompts | Responses invalid for one participant who accidentally misinterpreted the scale |
|  | Ik zit vol goede ideeën | I am full of good ideas | Not at all – reasonably – very much | All five prompts |  |
|  | Ik heb het gevoel te kort te schieten | I feel inadequate | Not at all – reasonably – very much | All five prompts |  |
|  | Mijn gedachten gaan snel | My thoughts are racing | Not at all – reasonably – very much | All five prompts |  |
|  | Ik kan snel schakelen | I am able to focus and switch easily | Not at all – reasonably – very much | All five prompts |  |
|  | Ik ben snel afgeleid | I’m distracted easily | Not at all – reasonably – very much | All five prompts |  |
|  | Ik heb zin om met anderen af te spreken | I feel like socializing | Not at all – reasonably – very much | All five prompts |  |
|  | Eigen vraag | Personal question | Not at all – reasonably – very much | All five prompts | Item differs across participants |
|  | Ik heb meer gegeten dan gewoonlijk | I have eaten more than usual | Not at all – reasonably – a lot more than usual | All five prompts | Too dependent on the occurrence of events |
|  | Sinds het vorige meetmoment heb ik gepiekerd | Since the previous prompt, I have worried | Not at all – reasonably – very much | All five prompts |  |
|  | Sinds het vorige meetmoment heb ik veel gecommuniceerd | Since the previous prompt, I have communicated a lot | Not at all – reasonably – very much | All five prompts | Too dependent on the occurrence of events |
|  | Sinds het vorige meetmoment heb ik me lichamelijk ingespannen | Since the previous prompt, I have been physically active | Not at all – reasonably – very much | All five prompts |  |
|  | Denk aan de meest opvallende gebeurtenis sinds het vorige meetmoment. Hoe heftig was deze gebeurtenis? | Think back on the most notable event since the previous prompt. How intense was this event? | Not at all – reasonably – very much | All five prompts | Too dependent on the occurrence of events |
|  | Is dit de laatste meting die u invult vandaag? | Is this the last assessment you complete today? | - Yes - No | All five prompts |  |
|  | 1. Ik heb vandaag veel afspraken gemaakt | I have made a lot of appointments today | Not at all – reasonably – a lot | If answered ‘yes’ on item 26 | Assessed on different timescale, yielding too few data points for analysis |
|  | Sinds het vorige meetmoment, wat deed ik? | Since the previous prompt, what have I been doing? (multiple options possible) | - Sleeping  - Household chores/groceries  - Working/studying  - Doing sports/walking/cycling  - Something relaxed (e.g., reading, TV)  - Hobby (e.g., gardening, music)  - A trip (e.g., into town, concert)  - Something together with others  - Something intimate (e.g., cuddling, sex)  - Engaging in self-care  - Resting/nothing  - On the way  - Something else | All five prompts | Not a continuous variable |
|  | Noteer hier eventuele opmerkingen. Noteer het ook als er iets gebeurd is dat invloed heeft op uw stemming. | Note observations here if any. Also note anything that may have influenced your mood. | Open entry | All five prompts (optional) | Not a continuous variable |

## Results of the sensitivity analyses for the window size

All early warning signal (EWS) analyses were run for window sizes of 1, 2, and 3 weeks. Supplementary Table 2 shows the predictive values, sensitivity, and specificity of the autocorrelation and standard deviation, averaged across all EMA diary items. The differences between the window sizes are quite small. One notable finding is that a three-week window is preferable when examining the autocorrelation prior to transitions to depression and mania. For the standard deviation, the two-week window was preferable.

| **Supplementary Table 2. Predictive values, sensitivity, and specificity of early warning signals, for three different window sizes, averaged across all EMA diary items.** | | | | | | | |
| --- | --- | --- | --- | --- | --- | --- | --- |
|  | **Depression** | | | **Mania** | | |  |
|  | **PPV** | **NPV** | **Sensitivity** | **PPV** | **NPV** | **Sensitivity** | **Specificity** |
| *Indicator: AR* |  |  |  |  |  |  |  |
| 1 week window | 43 | 65 | 25 | 45 | 70 | 25 | 78 |
| **2 week window** | **46** | **63** | **27** | **48** | **69** | **33** | **75** |
| 3 week window | 59 | 69 | 36 | 58 | 73 | 30 | 84 |
| *Indicator: SD* |  |  |  |  |  |  |  |
| 1 week window | 32 | 61 | 18 | 42 | 70 | 30 | 72 |
| **2 week window** | **27** | **58** | **23** | **41** | **71** | **38** | **67** |
| 3 week window | 18 | 38 | 15 | 38 | 68 | 30 | 67 |

*Abbreviations:* AR = autocorrelation, EMA = ecological momentary assessment (EMA), PPV = positive predictive value, NPV = negative predictive value, SD = standard deviation.

## Calculation of positive and negative predictive values, sensitivity, and specificity

The sensitivity of EWS reflects the probability of EWS, given that a transition is present. Sensitivity was calculated per indicator (autocorrelation, standard deviation), momentary state (*i*) and transition type (*t*; depression, mania) by dividing the number of EWS in momentary state *i* that anticipated transition *t* by the total number of transitions of type *t*. As an example, the autocorrelation in feeling tired anticipated four out of eight transitions towards depression, resulting in a sensitivity of .50 (i.e., 50%).

$${sensitivity}_{i,t}=\frac{{EWS}_{i,t}}{N_{t}}$$

The specificity of EWS reflects the probability of *no* EWS, given that there was no transition. This statistic was calculated by inspecting EWS in those individuals who did not experience a transition (N = 7). A specificity of 1 (i.e., 100%) indicates that EWS in this particular item were never found in individuals without transitions.

$${specificity}_{i}=1- \frac{{EWS}_{i}}{N}$$

Positive predictive values (PPVs) indicate the probability of a transition towards either mania or depression, given that EWS are detected. These values were calculated as follows:

$${PPV}_{i,t}=\frac{{sensitivity}_{i,t}*{prevalence}_{t}}{{sensitivity}_{i,t}*{prevalence}_{t}+\left( 1- {specificity}_{i} \right)*(1- {prevalence}_{t})}$$

In this formula, the prevalence of a transition of type *t* reflects the number of transitions of type *t* divided by the total number of transitions. In total, we identified 22 transitions, of which 8 (36%) involved a sudden increase in symptoms of depression, 7 (32%) involved an increase in manic symptoms, and 7 (32%) were simulated in individuals without transitions. The latter ‘non-transitions’ were used to estimate the specificity of EWS.

Negative predictive values (NPVs) indicate the probability that *no* transition will occur, given that EWS are *not* detected. These values were calculated as follows:

$${NPV}_{i,t}=\frac{{specificity}_{i}*{(1-prevalence}_{t})}{{(1-sensitivity}_{i,t})*{prevalence}_{t}+ {specificity}_{i}*(1- {prevalence}_{t})}$$

Note that the probability of *no* transition is defined by 1 minus the prevalence of a particular type of transition, resulting in 64% (depression) and 68% (mania), respectively. The prevalence of a transition can be thought of as a set-point: in absence of any insight in EWS, the probability that a specific individual will (not) experience a transition equals the average probability of (no) transitions. EWS can be considered informative if (1) their presence considerably heightens the risk of a future transition and (2) their absence lowers the probability that a transition will occur. Therefore, in Supplementary Table 3, we printed PPV and NPV values that exceeded the expected probabilities (${prevalence}_{t}$ or $1-{prevalence}_{t})$.

| **Supplementary Table 3. Predictive values, sensitivity, and specificity of early warning signals for transitions towards depression and mania.** | | | | | | | |
| --- | --- | --- | --- | --- | --- | --- | --- |
|  | **Depression** | | | **Mania** | | |  |
|  | **PPV** | **NPV** | **Sensitivity** | **PPV** | **NPV** | **Sensitivity** | **Specificity** |
| *Indicator: AR* |  |  |  |  |  |  |  |
| calm | 20 | 59 | 12 | 19 | 64 | 14 | 71 |
| cheerful | **100** | **74** | 38 | **100** | **71** | 14 | 100 |
| content | 23 | 44 | 38 | 9 | 42 | 14 | 29 |
| irritated | 20 | 59 | 12 | **41** | **73** | 43 | 71 |
| thoughts racing | **50** | **67** | 25 | **70** | **87** | 71 | 86 |
| distracted | 0 | 50 | 0 | 32 | 68 | 43 | 57 |
| physically active | **43** | **67** | 38 | 32 | 68 | 29 | 71 |
| socializing | 20 | 50 | 25 | 19 | 56 | 29 | 43 |
| down | 14 | 53 | 12 | 24 | 63 | 29 | 57 |
| inadequate | **43** | **67** | 38 | 19 | 64 | 14 | 71 |
| worry | 33 | 63 | 12 | **58** | **76** | 43 | 86 |
| tired | **67** | **75** | 50 | **48** | **72** | 29 | 86 |
| agitated | **50** | **67** | 25 | **48** | **72** | 29 | 86 |
| extremely well | **43** | **67** | 38 | 32 | 68 | 29 | 71 |
| full of ideas | **100** | **70** | 25 | **100** | **83** | 57 | 100 |
| focus switch | **100** | **74** | 38 | **100** | **75** | 29 | 100 |
| full of energy | **50** | **67** | 25 | **58** | **76** | 43 | 86 |
| *Indicator: SD* |  |  |  |  |  |  |  |
| calm | 33 | 63 | 12 | **58** | **76** | 43 | 86 |
| cheerful | 0 | 50 | 0 | 24 | 63 | 29 | 57 |
| content | 20 | 59 | 12 | **41** | **73** | 43 | 71 |
| irritated | 33 | 63 | 12 | **48** | **72** | 29 | 86 |
| thoughts racing | 14 | 53 | 12 | **44** | **81** | 71 | 57 |
| distracted | 0 | 56 | 0 | **41** | **73** | 43 | 71 |
| physically active | 27 | 55 | 38 | 26 | 62 | 43 | 43 |
| socializing | 14 | 53 | 12 | 24 | 63 | 29 | 57 |
| down | 20 | 59 | 12 | 19 | 64 | 14 | 71 |
| inadequate | 20 | 59 | 12 | 19 | 64 | 14 | 71 |
| worry | **100** | **70** | 25 | **100** | **75** | 29 | 100 |
| tired | 14 | 53 | 12 | 24 | 63 | 29 | 57 |
| agitated | 33 | 63 | 12 | **65** | **81** | 57 | 86 |
| extremely well | 20 | 59 | 12 | **48** | **78** | 57 | 71 |
| full of ideas | 25 | 57 | 25 | 32 | 68 | 43 | 57 |
| focus switch | 20 | 50 | 25 | 26 | 62 | 43 | 43 |
| full of energy | **60** | **71** | 38 | **65** | **81** | 57 | 86 |

*Abbreviations.* AR = autocorrelation, NPV = negative predictive value, PPV = positive predictive value, SD = standard deviation

## Effect of detrending

Given that detrending potentially affects the detection of EWS, we re-ran our analyses with linear instead of non-linear detrending. Specifically, within each window, we removed linear trends over time. This ensured that the detection of EWS was not affected by trends in mean symptom levels – which are to be expected from a clinical point of view, but were not the focus of this manuscript. Globally speaking, our results remained largely similar to what is reported in the main text. The autocorrelation was more sensitive and specific to transitions compared to the standard deviation. Further, the presence of EWS changed the probability of a transition in depression from 32 (prevalence of depressive transitions) to 49% (autocorrelation) and 30% (standard deviation) respectively (Supplementary Table 4). For transitions towards mania (prevalence 36%), EWS had positive predictive values of 49% (autocorrelation) and 43% (standard deviation). On the level of specific items, however, results varied substantially, reflected by a Cohen’s Kappa of 0.51 (autocorrelation) and 0.62 (standard deviation). Kappa values for individual items are reported in Supplementary Table 5. Our findings with both detrending methods can be found below (Supplementary Table 4). Results for windows containing 1 and 3 weeks of observations are available upon request.

| **Supplementary Table 4. Comparison of linear vs. non-linear detrending** | | | | | |
| --- | --- | --- | --- | --- | --- |
| **ID** | **Transition** | **non-linear detrending** | | **linear detrending** | |
|  |  | *AR* | *SD* | *AR* | *SD* |
| 1.1 | depression | 3 | 3 | 7 | 1 |
| 1.2 | depression | 5 | 1 | 4 | 4 |
| 2.1 | depression | 5 | 2 | 6 | 8 |
| 2.2 | depression | 0 | 4 | 3 | 4 |
| 3 | depression | 2 | 1 | 2 | 0 |
| 4 | depression | 10 | 3 | 7 | 3 |
| 5 | depression | 4 | 5 | 3 | 3 |
| 6.2 | depression | 7 | 3 | 5 | 6 |
| *mean* (±*SD*) |  | 4,50 (±3,07) | 2,75 (±1,39) | 4,63 (±1,92) | 3,63 (±2,56) |
| PPV (±*SD*) |  | 46 (±31) | 27 (±23) | 49 (±31) | 30 (±16) |
| NPV (±*SD*) |  | 63 (±9) | 58 (±6) | 66 (±6) | 60 (±6) |
| 10 | mania | 6 | 8 | 6 | 5 |
| 11 | mania | 9 | 6 | 5 | 5 |
| 6.1 | mania | 5 | 6 | 4 | 7 |
| 7.1 | mania | 0 | 8 | 6 | 11 |
| 7.2 | mania | 5 | 5 | 3 | 3 |
| 8 | mania | 3 | 6 | 6 | 5 |
| 9 | mania | 11 | 8 | 8 | 8 |
| *mean* (±*SD*) |  | 5,57 (±3,64) | 6,71 (±1,25) | 5,43 (±1,62) | 6,29 (±2,63) |
| PPV (±*SD*) |  | 48 (±30) | 41 (±22) | 49 (±31) | 43(±19) |
| NPV (±*SD*) |  | 69 (±10) | 71 (±7) | 72 (±6) | 70 (±6) |
| 12 | none | 2 | 4 | 2 | 6 |
| 13 | none | 7 | 6 | 5 | 4 |
| 14 | none | 5 | 6 | 5 | 4 |
| 15 | none | 1 | 8 | 1 | 10 |
| 16 | none | 6 | 6 | 1 | 2 |
| 17 | none | 4 | 4 | 4 | 3 |
| 18 | none | 5 | 3 | 5 | 6 |
| *mean* (±*SD*) |  | 4,29 (±2,14) | 5,29 (±1,70) | 3,29 (±1,89) | 5,00 (±2,65) |
| *Note*. Numbers refer to the number of EWS found for each individual, the average number of EWS found across individuals (mean), and the predictive values averaged across individuals and items (PPV, NPV).  *Abbreviations.* AR = autocorrelation, NPV = negative predictive value, PPV = positive predictive value, SD = standard deviation. | | | | | |

| **Supplementary Table 5. Cohen’s Kappa (agreement between EWS obtained by linear vs. non-linear detrending) for individual EMA items.** | | |
| --- | --- | --- |
| item | Kappa AR | Kappa SD |
| agitated | *0,77* | **0,88** |
| calm | 0,17 | *0,74* |
| cheerful | -0,18 | 0,58 |
| content | 0,39 | 0,54 |
| distracted | 0,31 | *0,64* |
| down | *0,77* | 0,58 |
| extremely well | 0,58 | *0,79* |
| focus switch | 0,58 | **0,81** |
| full of energy | 0,49 | 0,50 |
| full of ideas | **0,88** | *0,79* |
| inadequate | 0,58 | 0,35 |
| irritated | **1,00** | 0,49 |
| physically active | **1,00** | *0,63* |
| socializing | 0,04 | 0,50 |
| thoughts racing | 0,47 | *0,62* |
| tired | 0,09 | 0,11 |
| worry | 0,47 | *0,74* |
| *Note*. Numbers in bold reflect strong agreement (Kappa > 0.8), numbers in italic reflect moderate agreement (Kappa > 0.6 (4)). The negative Kappa value for feeling cheerful (AR) means that agreement was lower than what would be expected from chance.  *Abbreviations.* AR = autocorrelation, EMA = ecological momentary assessment, EWS = early warning signal, SD = standard deviation. | | |

**Supplementary Figure 3. Illustration of positive and negative predictive values across items, where EWS were computed using linear detrending (window = 2 weeks). For feeling inadequate, predictive values for the SD (transition towards depression) could not be computed because sensitivity was 0 and specificity was 1 (white tiles). This figure serves to illustrate that a different detrending method affected our results on the level of specific items (*i.e.,* predictive values of particular items), but not on a more global level (*i.e.,* average predictive values).**


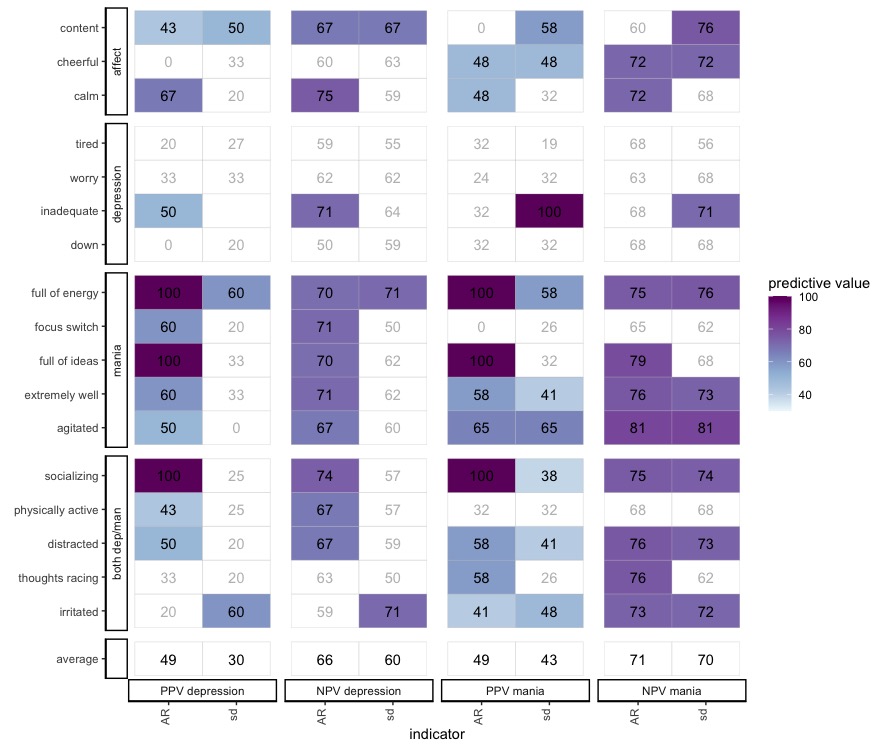


*Abbreviations.* AR = autocorrelation, EWS = early warning signal, NPV = negative predictive value, PPV = positive predictive value, SD = standard deviation.

**Supplementary Figure 4. EWS detected in the AR (upper/left plot) and SD (lower/right plot) for specific individuals (y-axis), who sometimes experienced more than one transitions (denoted by digits, with the lowest digit corresponding to the first transition). EWS were detected using moving window analyses (window = 2 weeks). Within each window, we used linear detrending to prevent confusing trends in mean levels for trends in the autocorrelation or variance. Results are comparable, but not exactly similar, to our main findings. Averaged across individuals, however, the method used for detrending does not impact the presence and predictive capacity of EWS.**


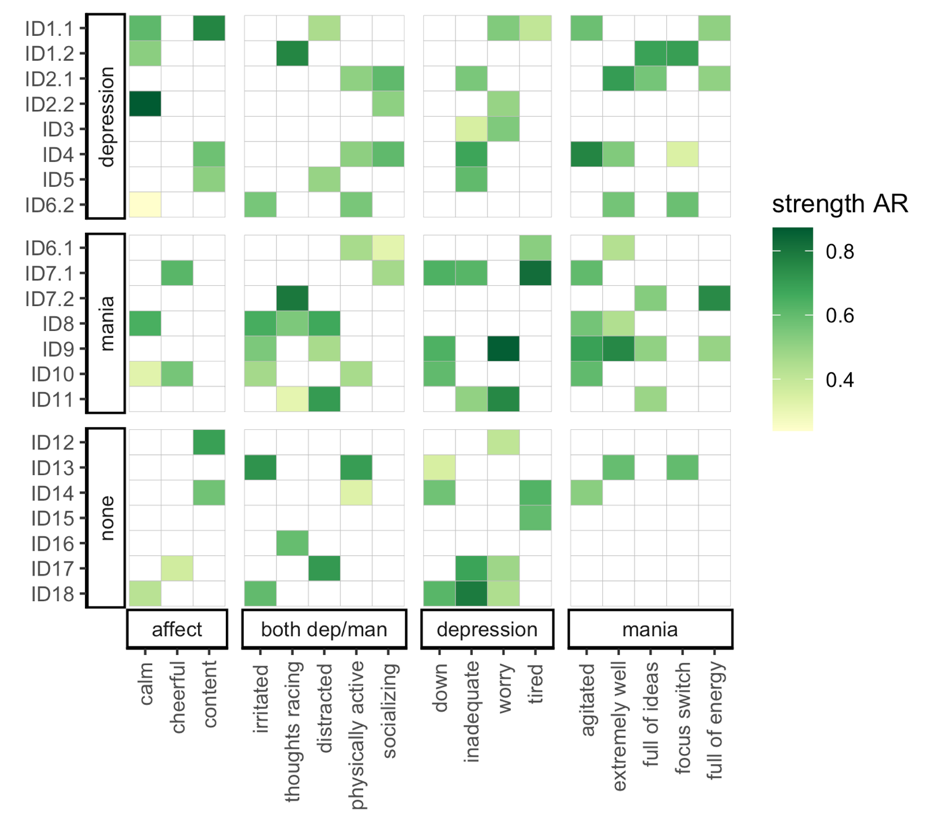


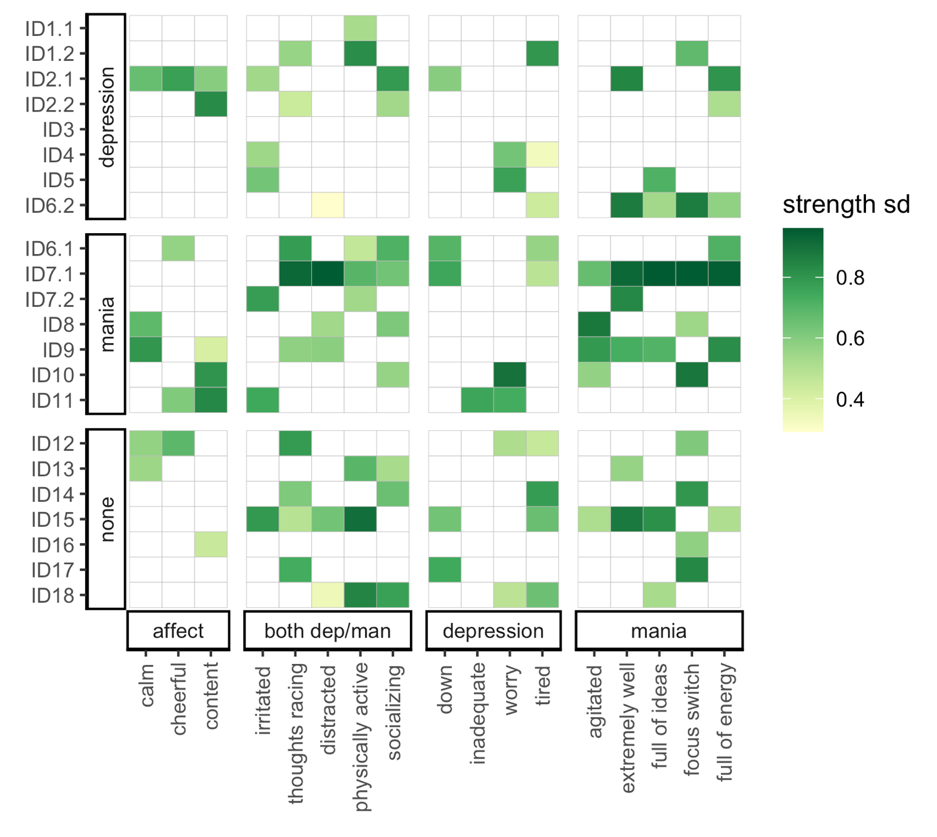


*Abbreviations.* AR = autocorrelation, EWS = early warning signal, SD = standard deviation.

## Agreement between EWS

Agreement between the autocorrelation (AR) and standard deviation (SD) as EWS for transitions in bipolar disorder was absent, reflected by a Cohen’s Kappa of 0.00318. In Supplementary Figure 5, we illustrated the agreement between the AR and SD for towards depression (left) and mania (right). It can be seen that, out of the 54 EWS that were found, only 4 occurred for the same person and EMA item (namely: ID 6, second transition: feeling extremely well and focus switching, ID 3: feeling inadequate, ID 5: socializing).

**Supplementary Figure 5. Agreement between EWS.**


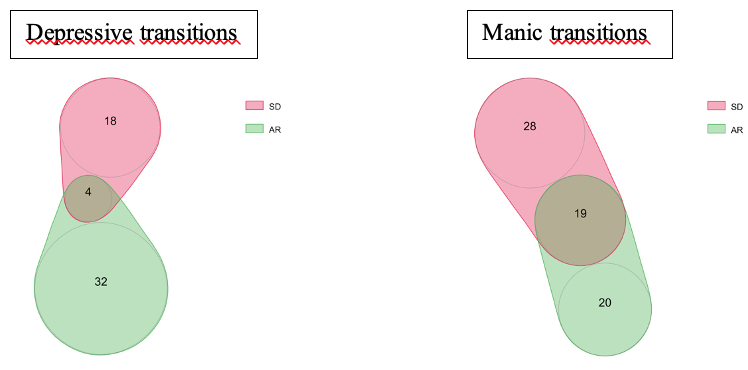


References

1. Knapen SE. Rhythm & Blues: Chronobiology in the Pathophysiology and Treatment of Mood Disorders: Rijksuniversiteit Groningen; 2019.

2. van der Krieke L, Jeronimus BF, Blaauw FJ, Wanders RBK, Emerencia AC, Schenk HM, et al. HowNutsAreTheDutch ((HoeGekIsNL): A crowdsourcing study of mental symptoms and strengths. International Journal of Methods in Psychiatric Research. 2016;25(2):123.

3. Tyler E, Jones S, Black N, Carter L-A, Barrowclough C. The relationship between bipolar disorder and cannabis use in daily life: An experience sampling study. PLoS One. 2015;10(3).

4. McHugh ML. Interrater reliability: the kappa statistic. Biochemia medica. 2012;22(3):276-82.
